# Dynamics of Viral Abundance and Diversity in a *Sphagnum*-Dominated Peatland: Temporal Fluctuations Prevail Over Habitat

**DOI:** 10.3389/fmicb.2015.01494

**Published:** 2016-01-06

**Authors:** Flore Ballaud, Alexis Dufresne, André-Jean Francez, Jonathan Colombet, Télesphore Sime-Ngando, Achim Quaiser

**Affiliations:** ^1^UMR CNRS 6553 ECOBIO, Université de Rennes 1Rennes, France; ^2^Université Clermont Auvergne, Université Blaise PascalClermont-Ferrand, France; ^3^CNRS, UMR 6023, Laboratoire Microorganismes: Génome et EnvironnementAubière, France

**Keywords:** fen, bog, metaviromes, prokaryotes, ecological succession, microbial interactions

## Abstract

Viruses impact microbial activity and carbon cycling in various environments, but their diversity and ecological importance in *Sphagnum*-peatlands are unknown. Abundances of viral particles and prokaryotes were monitored bi-monthly at a fen and a bog at two different layers of the peat surface. Viral particle abundance ranged from 1.7 x 10^6^ to 5.6 x 10^8^ particles mL^-1^, and did not differ between fen and bog but showed seasonal fluctuations. These fluctuations were positively correlated with prokaryote abundance and dissolved organic carbon, and negatively correlated with water-table height and dissolved oxygen. Using shotgun metagenomics we observed a shift in viral diversity between winter/spring and summer/autumn, indicating a seasonal succession of viral communities, mainly driven by weather-related environmental changes. Based on the seasonal asynchrony between viral and microbial diversity, we hypothesize a seasonal shift in the active microbial communities associated with a shift from lysogenic to lytic lifestyles. Our results suggest that temporal variations of environmental conditions rather than current habitat differences control the dynamics of virus-host interactions in *Sphagnum*-dominated peatlands.

## Introduction

Viruses are present in virtually all ecosystems, and are considered to be the most abundant biological entities in the biosphere (Suttle, 2005). Through lytic and lysogenic life cycles, viruses affect the metabolism and abundance of their cellular hosts from all three domains of life (synthesis in) (Kirchman, 2012), impacting the diversity and the structure of microbial communities (Middelboe, 2000; Suttle, 2007; Sime-Ngando, 2014). Because viruses function as a top-down control on microbial production (Pradeep Ram et al., 2011; Chow et al., 2014), they affect biogeochemical cycles through the release of substantial amounts of organic carbon and nutrients in the environment (Middelboe and Lyck, 2002). While resources released by this viral shunt can be reused to sustain microbial biomass, it typically results in a reduction of the overall flow of organic matter and energy towards higher trophic levels (Fuhrman, 1999; Middelboe and Lyck, 2002; Ankrah et al., 2014). Besides the viral-shunt, a significant proportion of the released viruses represent a consequential amount of labile organic matter that can be readily decomposed (Dell’Anno et al., 2015). Viral lysis, along with bacterial grazing are important processes in microbial succession (Fierer et al., 2010), suggesting that coupled viral-host interactions can influence ecosystem-level carbon cycling, depending on the activity of cells and on the balance between lytic and lysogenic strategies. Consequently, considering viral ecology is critical to understanding ecosystem functioning.

Peatlands are a globally relevant component of the carbon cycle, storing a quarter of global soil carbon and more carbon than all vegetation (Yu, 2012; Turetsky et al., 2015). Boreal and temperate peatlands are often dominated by peat forming mosses, belonging to the *Sphagnum* genus. Many *Sphagnum-*dominated peatlands develop from a nutrient-rich, non-acidic fen (minerotrophic fen) during early stages, to a nutrient-poor acidic bog (rain-fed ombrotrophic bog) (Rydin and Jeglum, 2006). During this development process, peatlands accumulate large stocks of partly decomposed organic matter as peat. This accumulation is a consequence of the long-term imbalance between carbon uptake from photosynthesis and carbon losses *via* respiration, methanogenesis and DOC leaching, which results in the preservation of up to 15 % of the net primary production as peat (Clymo, 1984; Moore, 1987; Francez and Vasander, 1995; Roulet et al., 2007). The combination of abiotic conditions, such as low temperature, low pH, high soil water content and poor nutrient availability, as well as biotic factors (*Sphagnum* litter quality, allelopathy) constrains microbial activities, limiting decomposition and mineralization (Clymo, 1984; Rydin and Jeglum, 2006). Accumulation of organic matter over long time periods leads to stratification with a deep permanently anoxic catotelm in contact with underlying bedrock, an intermediate mesotelm layer that is periodically anoxic and oxic and the predominantly oxic acrotelm at the surface (Clymo and Bryant, 2008). The heterogeneity of peat, the variety of dynamic stages and sources of water and nutrients provide a large panel of habitats and niches for a broad diversity of microorganisms transforming different carbon substrates through aerobic and anaerobic pathways of decomposition (Juottonen et al., 2005; Artz, 2009; Andersen et al., 2010; Tveit et al., 2012).

Understanding of microbial diversity in peatlands, especially in regards to spatio-temporal patterns, is currently limited. The development of cultivation-independent approaches such as metagenomics provide a powerful tool to investigate microbial taxa and associated protein-coding genes across ecosystems (Lynch et al., 2004; Vandenkoornhuyse et al., 2010). More recently, sequencing of viral metagenomes (hereafter metaviromes) has dramatically expanded understanding of viral diversity, particularly in marine and freshwater environments (Comeau et al., 2008; Lopez-Bueno et al., 2009; Rohwer and Thurber, 2009; Roux et al., 2012; Smith et al., 2013; Labonté and Suttle, 2013; Wommack et al., 2015) compared to soil ecosystems (Kimura et al., 2008; Zablocki et al., 2014; Adriaenssens et al., 2015). Most analyses have focused on cross-biomes comparisons, while spatio-temporal dynamics remain poorly documented (Pagarete et al., 2013; Chow et al., 2014). Several viral marker genes, conserved within particular viral families, are considered to be reliable for taxonomic affiliation (Roux et al., 2011; Sakowski et al., 2014). Nevertheless, none of these marker genes are ubiquitous among viruses and they are far from the high level of conservation found in rRNA genes that are used for the classification of organisms from the three kingdoms. While several studies have investigated the diversity of microbial communities in peatlands (Dedysh et al., 2006; Artz et al., 2007; Peltoniemi et al., 2009; Dedysh, 2011; Mackelprang et al., 2011; Bragina et al., 2012; Serkebaeva et al., 2013; Mondav et al., 2014; Tveit et al., 2014; Hultman et al., 2015; Nunes et al., 2015) viral diversity in *Sphagnum*-dominated peatlands remains largely unknown and basic knowledge of virus ecology in these ecosystems is still lacking (Quaiser et al., 2015). In view of the importance of viruses in structuring and regulating prokaryotic communities and the implication of the latter in the carbon sink function of peatland; it is essential to understand the role of viruses in the dynamics of the microbial communities in this ecosystem.

In order to characterize virus ecology in a temperate *Sphagnum-*dominated peatland, we combined and integrated different approaches to study the spatio-temporal patterns of viral abundance and diversity. The goals of this work were: (1) to compare the seasonal abundance of viruses and prokaryotes at two different layers of the peat surface corresponding to a stratified analysis of acrotelm, the most active layer, of fen and bog; (2) to identify abiotic controls on abundance and distribution of these viruses and prokaryotes; and (3) to characterize the viral diversity over an annual cycle.

## Materials and Methods

### Site Description

Peat samples were collected at Les Pradeaux (3°55E; 45°32N), a *Sphagnum*-dominated peatland situated in the French Massif Central at an altitude of 1 350 m. The fen is dominated by *Sphagnum fallax, Carex rostrata, Eriophorum angustifolium*, and *Menyanthes trifoliata*, while the bog is mainly colonized by *Sphagnum magellanicum, Sphagnum capillifolium, Andromeda polifolia, Vaccinium oxycoccos*, and *Eriophorum vaginatum*.

### Sampling Strategy and Experimental Design

Twelve field sessions were organized between May 2010 and November 2012 (Supplementary Table S1): One in May 2010, four in 2011 (June, August 12th, August 24th and October), and seven in 2012 (March, May, June, July, August, September, and November).

For each field session, three peat profiles (replicates) were analyzed in the fen and the bog. In order to avoid disturbance during the extraction of peat and water, sampling for viral counts and metagenomes followed a 3-step progressive cutting from surface to depth. The 0-5 cm layer corresponding to the living *Sphagnum* carpet (capitulum and “green” stems and leaves) was cut off and carefully removed. The 5-10 cm layer (called “upper surface layer” thereafter) was then cut off and a fraction of peat matrix was harvested and stored in a 50 mL tube for metagenome production. From the remaining undisturbed peat, pore-water was expressed for viral and prokaryote counts. The same method was applied to the 10-15 cm layer (called “lower surface layer” thereafter). For metaviromes production as well as for physico-chemical analysis pore-water was expressed from the remaining peat surface layers combined. At least 200 mL of pore-water were necessary for metavirome production.

### Prokaryote and Viral-Like Particle Abundances

For each layer of each sampling point, at least 20 mL of peat water was extracted, prefiltered at 125 μm, and fixed in Glutaraldehyde (2%, Grade II, Sigma-Aldrich). Prokaryote abundance (PA) and viral particle abundance (VPA) were obtained using flow cytometry (BD Accuri C6) (Sime-Ngando et al., 2008).

### Physico-Chemical Parameters

Peat temperature was measured directly in the peat profiles at 5, 10, and 15 cm under the *Sphagnum capitula* layer. Conductivity, pH and oxygen were measured in the field with filtered (125 μm) peat water. Dissolved organic carbon (DOC) and anion concentrations (nitrate, sulfate, and acetate) were measured at the “Biogeochemical Analysis” platform (ECOBIO and GEOSCIENCES - OSU Rennes), following water filtration at 0.2 μm (Whatman), using a Bioritech DOC Analyser and a Dionex Analyzer (**Table 1**).

**Table 1:** Physico-chemical parameters measured in the peat samples from 2012.

		Temperature (C°)	Water-table (cm)	pH	Conductivity (μS cm^-1^)	Sulfate (mg.L^-1^)	Nitrate (mg.L^-1^)	Oxygen (mg.L^-1^)	DOC (ppm)
**FEN**	March	14.5 (1.3)	1 (0)	4.7 (0.2)	17.6 (4.2)	0.70 (0.14)	0.1 (0.01)	9.54 (0.55)	15.6 (3.1)
	May	14.3 (0.6)	0.8 (1.8)	4.6 (0.1)	16.1 (1.1)	0.67 (0.07)	0.40 (0.42)	6.33 (0.02)	19.7 (2.2)
	June	14 (0.5)	-0.2 (0.6)	6.1 (0.1)	16.8 (4.8)	0.69 (0.06)	0.99 (0.72)	6.57 (0.90)	25.3 (3.3)
	July	13.8 (1.6)	-5.6 (1.2)	6.6 (0.2)	12.8 (0.7)	1.08 (0.07)	1.04 (0.67)	9.61 (0.84)	18.4 (1.2)
	August	15 (1.0)	-10 (1)	4.6 (0.1)	97.7 (4.8)	1.85 (0.38)	1.45 (0.07)	0.44 (0.02)	NA
	September	10.1 (1.4)	-7.3 (0.6)	4.6 (0.1)	34.6 (8.2)	1.64 (0.10)	1.40 (0.03)	1.28 (1.11)	38.4 (2.5)
	November	5 (4.4)	1 (0)	4.6 (0.1)	56.9 (32.8)	1.67 (0.09)	1.66 (0.09)	9.73 (0.35)	NA

**BOG**	March	11 (3.0)	-12.3 (2.5)	4.7 (0.1)	44.2 (23.7)	1.24 (0.35)	0.94 (0.09)	7.28 (0.20)	19.5 (0.7)
	May	13.7 (0.8)	-10.3 (1.5)	4.4 (0.2)	42.5 (8.1)	2.20 (0.41)	1.30 (0.21)	5.69 (0.56)	27.9 (5.3)
	June	12.8 (0.8)	-16.2 (1.6)	4.4 (0.3)	45.3 (13.2)	0.94 (0.19)	0.57 (0.66)	5.83 (0.49)	49.7 (9.1)
	July	13.8 (0.3)	-20.3 (2.1)	4.5 (0.1)	29.5 (3.4)	1.46 (0.09)	0.1 (0.01)	6.12 (0.34)	17.8 (1.3)
	August	16.8 (1.4)	-23.7 (2.1)	4.2 (0.1)	61.8 (2.6)	2.28 (0.17)	1.67 (0.17)	1.77 (1.68)	NA
	September	10.7 (0.6)	-21.3 (2.1)	4.3 (0.1)	62.8 (25.3)	3.01 (0.60)	0.1 (0.01)	0.30 (0.07)	47.9 (12.3)
	November	2.7 (0.6)	-22 (2.0)	4.1 (0.1)	47.5 (18.3)	2.13 (0.44)	0.05 (0.02)	10.96 (0.58)	NA

*Mean (±SD) (*n* = 3), DOC: Dissolved Organic Carbon; NA: not available.*

### Metavirome and Metagenome Production

To get enough material fen and bog viral communities were sampled from pore water combining the upper and lower surface layers. Samples were recovered at four dates (Supplementary Table S1, **Table 2**): 07 June 2011 (vFen_June11, vBog_June11; along with peat for a study of the microbial communities), 12 August 2011 (vFen_Aug11, vBog_Aug11), 12 October 2011 (vFen_Oct11, vBog_Oct11), and 23 March 2012 (vFen_Mar12, vBog_Mar12). Due to low water content of the peat, only one sample was collected for each dynamic stage for the three first dates. In March 2012, the fen and bog were sampled in biological triplicates (vFen_Mar12_A, B, C; vBog_Mar12_A, B, C). In total we prepared six fen and six bog metaviromes. *Sphagnum* water was prefiltered at 125 μm. Viruses were concentrated using PEGylation (Colombet et al., 2007). Viral concentrates were filtered through a 0.20 μm filter (Minisart, Sartorius) and diluted 5x in H_2_O (Sigma) to a volume of 5 mL. Remaining non-viral DNA was digested with 10 U DNAse RQ1 RNAse free (Promega) at 37°C for 1 h. Viral DNA was extracted as described previously (Quaiser et al., 2015). DNA quality was checked with the High Sensitivity DNA kit on a 2100 Bioanalyzer (Agilent). Whole genome amplification (WGA) was applied in triplicate for each sample using GenomiPhi V2 (GE Healthcare) following manufacturers’ instructions and the triplicates were pooled.

**Table 2:** Main characteristics of the 12 fen and bog metaviromes.

Date	June 2011		August 2011		October 2011		March					
Origin	Fen	Bog	Fen	Bog	Fen	Bog	Fen			Bog		
Sample	vFen_June11	vBog_June11	vFen_Aug11	vBog_Aug11	vFen_Oct11	vBog_Oct11	vFen_Mar12_A	vFen_Mar12_B	vFen_Mar12_C	vBog_Mar12_A	vBog_Mar12_B	vBog_Mar12_C
Metavir Id	1368	1369	1373	1374	1375	1376	1377	1378	1379	1380	1382	1370
No. of sequences > 250 pb	90 983	36 165	107 181	171 483	67 303	105 296	75 274	62 492	78 169	114 591	122 251	65 701
Average size (bp)	415	418	416	415	409	413	415	416	416	416	414	415
Total nucleotides (Mbp)	37.8	16.4	44.65	71.24	27.55	43.56	31.27	26.05	32.58	47.76	50.71	27.33
No. rRNA	0	0	1	3	0	22	8	3	11	9	7	5
No. of tRNA	50	40	207	249	5	245	119	138	77	109	101	272
Hits to KO_KEGG	19	16	111	151	37	1134	314	132	957	1115	310	76
Hits to COG	69	59	113	276	46	1053	535	278	873	1011	550	167
% “No Hit”	91.30%	88.30%	92.99%	92.83%	94.10%	95.47%	93.64%	94.09%	94.08%	91.90%	89.52%	90.01%
No. shared sequences	1027	109	1492	817	8335	3792	329	195	1786	66	336	42
% shared	1.13%	0.30%	1.39%	0.48%	12.38%	3.60%	0.44%	0.31%	2.28%	0.06%	0.27%	0.06%

*% No hit: percentage of sequences that did not match against the “RefSeqVirus” database (tBLASTx, *e*-value 10^-7^).*

*shared: sequences that are common between at least one metagenome and one metavirome (Compareads 1.2.2., *t* = 4, *k* = 33).*

Subsequent pyrosequencing was performed on a GS FLX system (454 Life Sciences, Roche, Branford, CT, USA) at the “Functional and Environmental Genomics” platform (OSU, Rennes, France). Roche/454 filtering tools and stringent filters were used to ensure the highest sequence quality and to remove artificial replicates of sequences and sequences smaller than 250 bp as shown previously (Quaiser et al., 2014).

For the metagenomes, 12 un-pressed peat matrix samples, distinguishing bog/fen, upper and lower surface layers, each in triplicates, collected on 07 June 2011, underwent DNA extraction, pyrosequencing as well as size and quality trimming (Supplementary Table S2) as described previously (Quaiser et al., 2014). Briefly, 2 g of peat matrix were lysed in 15ml of extraction buffer containing 4% cetyltrimethylam- monium bromide (CTAB), 0.5% polyvinylpyrrolidone (PVP, Sigma-Aldrich), 0.7 M NaCl, 100 mM potassium phosphate (pH 6.8), 20 mM EDTA (pH 8.0), 1% beta-mercaptoethanol, 1 M guanidin thiocyanate and incubated at 65°C for 30 min. Homogenization was obtained by vigorous vortexing every 5 min during 1 min in the presence of glass beads. One volume of chloroform-isoamylalcohol (24:1) was added, vortexed for 1 min and incubated at room temperature for 5 min. The samples were centrifuged at 4000 *g* for 15 min at 4°C and the aqueous phases were transferred to new tubes. Binding conditions for silica-based RNA extraction were adjusted, applied on Nucleo Spin RNA II kit columns and subsequent purification was performed following the instructions of the manufacturer (Macherey-Nagel). DNA was nebulized to fragments of about 700 bp. The DNA was purified with Agencourt AMPur XP magnetic beads (Beckman-Coulter). DNA fragmentation quality was checked with the High sensitivity DNA kit on a 2100 Bioanalyzer (Agilent). Subsequent library construction and pyrosequencing was performed in technical duplicates on a GS FLX system (454/Roche) at the “Functional and Environmental Genomics” platform (OSU, Rennes, France). Roche/454 filtering tools and stringent filters developed locally were used to ensure the highest possible sequence quality and to suppress artificial replicates of sequences as well as sequences smaller than 250 bp.

The metaviromes are available under the Metavir IDs (http://metavir-meb.univ-bpclermont.fr/): 1368, 1369, 1370, 1373, 1374, 1375, 1376, 1377, 1378, 1379, 1380, and 1382 (project: VIRTOU). In addition, pyrosequencing reads reported in this publication have been deposited in the ENA Sequence Read Archive under the study accession number PRJEB11420 (metaviromes) and PRJEB11421 (metagenomes).

### Metavirome Analysis

After pyrosequencing, sequence quality and size trimming, we obtained 481 402 and 615 487 sequences with an average length of 415 bp from fen and bog, respectively (**Table 2**). The quality of the virome extraction process was assessed by determining the amount of rRNA and tRNA sequences using Meta_RNA that identifies SSU and LSU rRNAs from the three kingdoms (Huang et al., 2009) and tRNAscan-SE (Lowe and Eddy, 1997). In total 69 rRNA (0.0063%) and 1681 tRNA (0.15%) sequences were identified indicating an insignificant level of potential contamination of microbial DNA. To avoid misinterpretation of the results, these sequences were excluded in subsequent analysis.

The viral diversity was analyzed using Metavir (Roux et al., 2014) and the sequences were subjected to tBLASTx (Altschul et al., 1997) against the NCBI RefSeqVirus database (*e*-value 10^-7^). Taxonomic assignment of the sequences was determined with MEGAN (Huson et al., 2007). Several accompanying tools were used on the Galaxy/Genouest bioinformatics platform (Le Bras et al., 2013).

To estimate the level of similarity between the viral communities, the proportion of similar sequences of each pair of metaviromes was computed with Compareads 1.2.2 (Maillet et al., 2012). Using this software, two sequences are considered to be similar if they present a defined number (*t*) of identical *k*-mers (*k*). To calibrate this analysis we tested 3 different numbers (*t* = 2; 4; 10) of identical 33mers (*k* = 33 nt). The most reliable results were obtained using four identical 33mers, parameters that were used for further analyses computed with Compareads. Compareads output is a percentage of similarity between a pair of metaviromes. These percentages were used to build a distance matrix, on which hierarchical clustering was performed using the R package pvclust package (Suzuki and Shimodaira, 2006) (distance = “correlation”, method = “average”).

Comparisons made with Compareads give a global estimate of the similarity between the metaviromes. In order to take into account the diversity of sequences within each metavirome, we analyzed the qualitative distribution (presence-absence) of clusters of highly similar sequences in the 12 metaviromes. This second analysis also allowed removal of potential bias due to the variation of the number of sequences obtained for each metavirome. Sequences from the 12 metaviromes were clustered using CD-HIT-EST (Huang et al., 2010) (*c* = 0.95; *n* = 8). Clustering results were used to compute Sørensen dissimilarity between pairs of metaviromes using MOTHUR (Schloss et al., 2009). Hierarchical clustering (pv-clust package) was used to represent compositional relatedness between metaviromes from the matrix of Sørensen dissimilarities. Clusters of sequences were split into different categories according to the amount of sequences they contained and the same analysis was performed for each size category.

### Analysis of the Sequences Shared by a Metagenome and a Metavirome

In order to find viral sequences in the metagenomes, and to analyze the link between the viral and microbial communities, sequences shared by at least one metagenome and one metavirome were retrieved using Compareads (*k* = 33, *t* = 4), and clustered using CD-HIT-EST (see “Metaviromes Analysis”). To ensure that this selection of sequences did not alter the compositional patterns observed for the total metaviromes, Sørensen dissimilarity was calculated with these clusters for the “shared” sequences originating from the metaviromes. Correspondence Analysis (CA) was performed on the whole dataset of shared sequences and the sample dissimilarities carried on the two first axes was represented with a hierarchical clustering (ade4 package) (Dray and Dufour, 2007). Taxonomic assignment of the sequences was obtained using Metavir tBLASTx output (*e*-value 10^-7^) (Roux et al., 2014), and analyzed with MEGAN (Huson et al., 2007).

### Statistical Analysis

Due to non-homogeneity of variance, one-factor Kruskal-Wallis tests were used on PA, VPA, and VPR in order to detect differences between sites (fen vs. bog), layers (upper vs. lower) and sampling dates. A principal component analysis (PCA) was performed on the physico-chemical dataset taking into account samples with available DOC (ade4 package) (Dray and Dufour, 2007). The first component was associated with the fluctuation of the physico-chemical variables through the habitats and seasons. We used the sample coordinates on the first component as a variable representing the spatio-temporal gradient (Legendre and Legendre, 1998; Ramette, 2007). Then potential relations between the gradient and log transformed PA, VPA, and VPR were tested using linear regression. All statistical analyses were performed using the open-source statistical software R (version 2.14) (R Development Core Team, 2013).

## Results

### Viral and Prokaryotic Bundance

**FIGURE 1:**
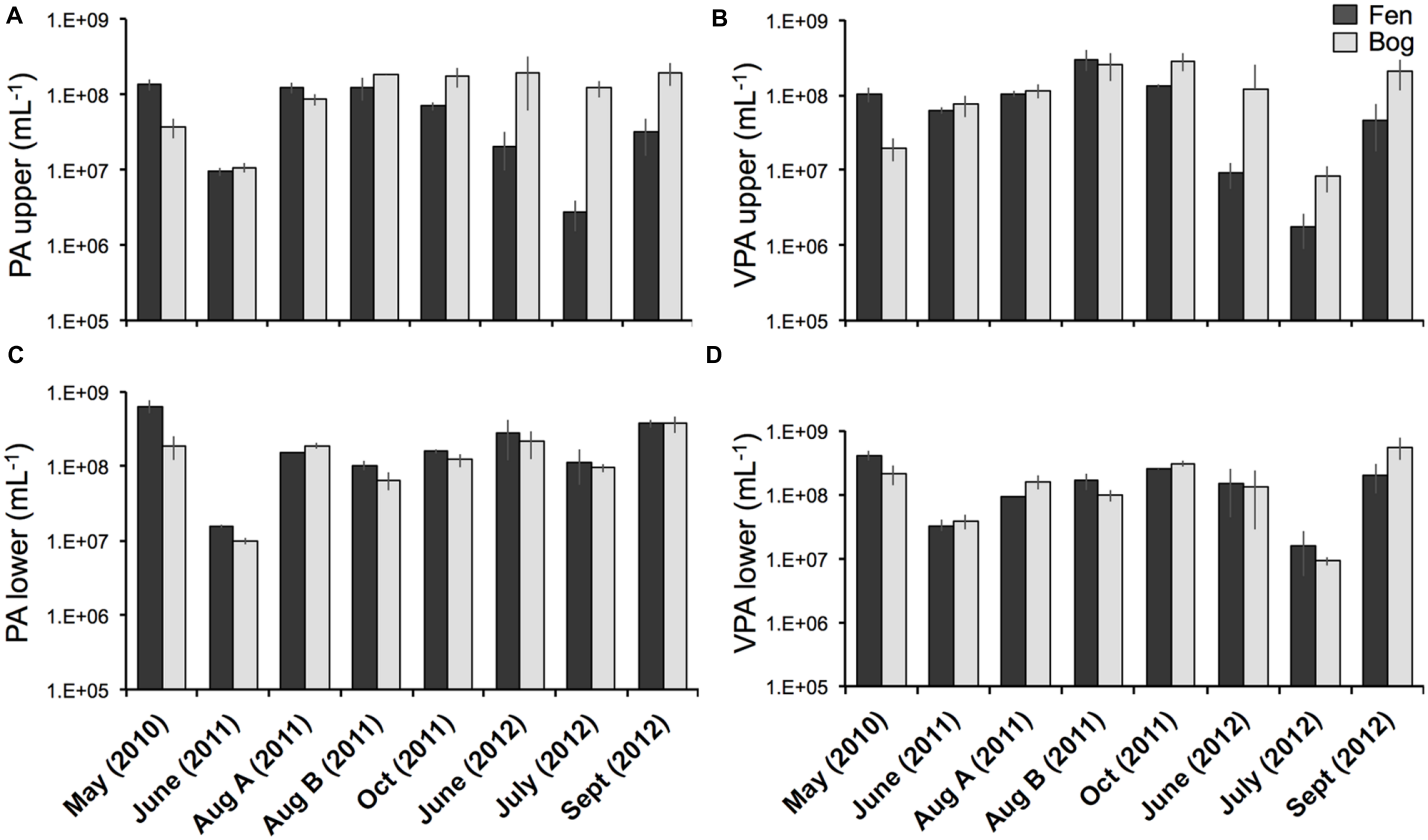
**Prokaryote abundance (PA) and viral particle abundance (VPA) from the fen and bog of the *Sphagnum-*dominated peatland. (A)** PA at the upper layer (5-10 cm), **(B)** PA at the lower layer (10-15 cm), **(C)** VPA at the upper layer, **(D)** VPA at the lower layer. Bars represent standard deviations (*N* = 3).

**FIGURE 2:**
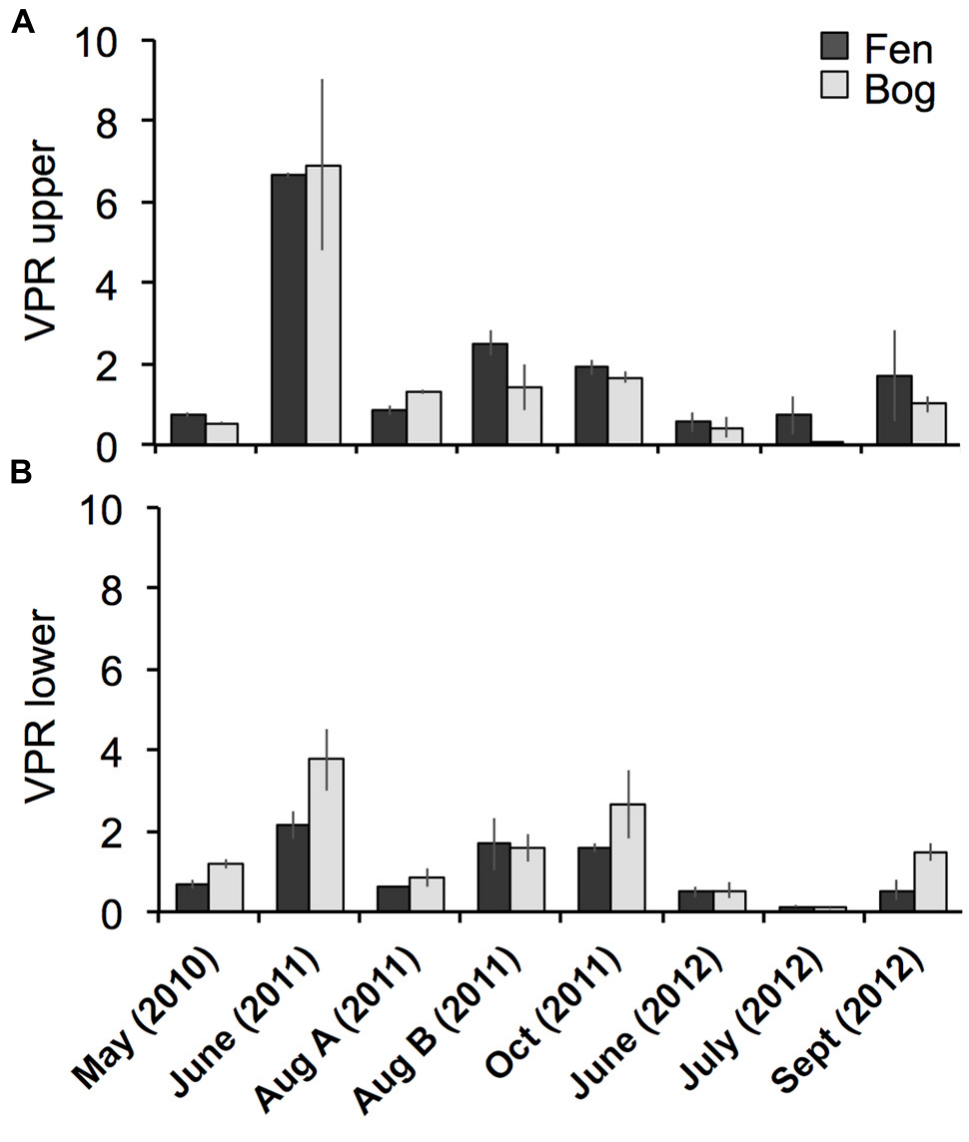
**Comparison of the virus to prokaryote ratio (VPR) of the *Sphagnum*–dominated peatland.** Bars represent standard deviations (*N* = 3).

Viral particle abundance and PA were investigated for the two peatland development stages over 2 years (**Figure 1**) aiming for the detection of spatial trends in the abundance of biological entities. VPA ranged from 1.7 ± 0.9 × 10^6^ (fen upper layer, July 2012) to 5.6 ± 2.1 x 10^8^ particles mL^-1^ (bog lower layer, September 2012), and PA ranged from 2.8 ± 1.2 × 10^6^ (fen upper layer, July 2012) to 6.3 ± 1.3 x 10^8^ cells mL^-1^ (fen lower layer, May 2010). VPA and PA were significantly correlated (Spearman, *r* = 0.76; *P* < 10^-15^; *N* = 95). We did not observe significant differences in PA and VPA between fen and bog; however, we detected significant variations with time (PA: KW-test, *P* = 1.8 × 10^-5^, *N* = 95; VPA: KW-test, *P* = 1.1 × 10^-8^, *N* = 95). While PA was significantly higher at the lower layer (average abundance of 9.5 ± 7.1 × 10^7^ cells mL^-1^ at the upper layer and 1.9 ± 1.6 × 10^8^ cells mL^-1^ at the lower layer) (KW-test, *P* < 0.05, *N* = 95), VPA did not differ significantly with depth (KW-test, *P* = 0.056, *N* = 95). Nevertheless, VPA followed a similar trend, with higher average abundances at lower layer compared with the upper layer (1.8 ± 1.6 × 10^8^ particles mL^-1^ vs0 1.2 ± 1.1 × 10^8^ particles mL^-1^, respectively). The virus to prokaryote ratio (VPR) differed by sampling date (**Figure 2**) (KW-test, *P* < 0.01, *N* = 95). No significant differences were observed between development stages or sampling depths. The highest VPR, measured in June 2011 was due to low PA rather than to high VPA.

### Link Between Abiotic Variables, VPA and PA

**FIGURE 3:**
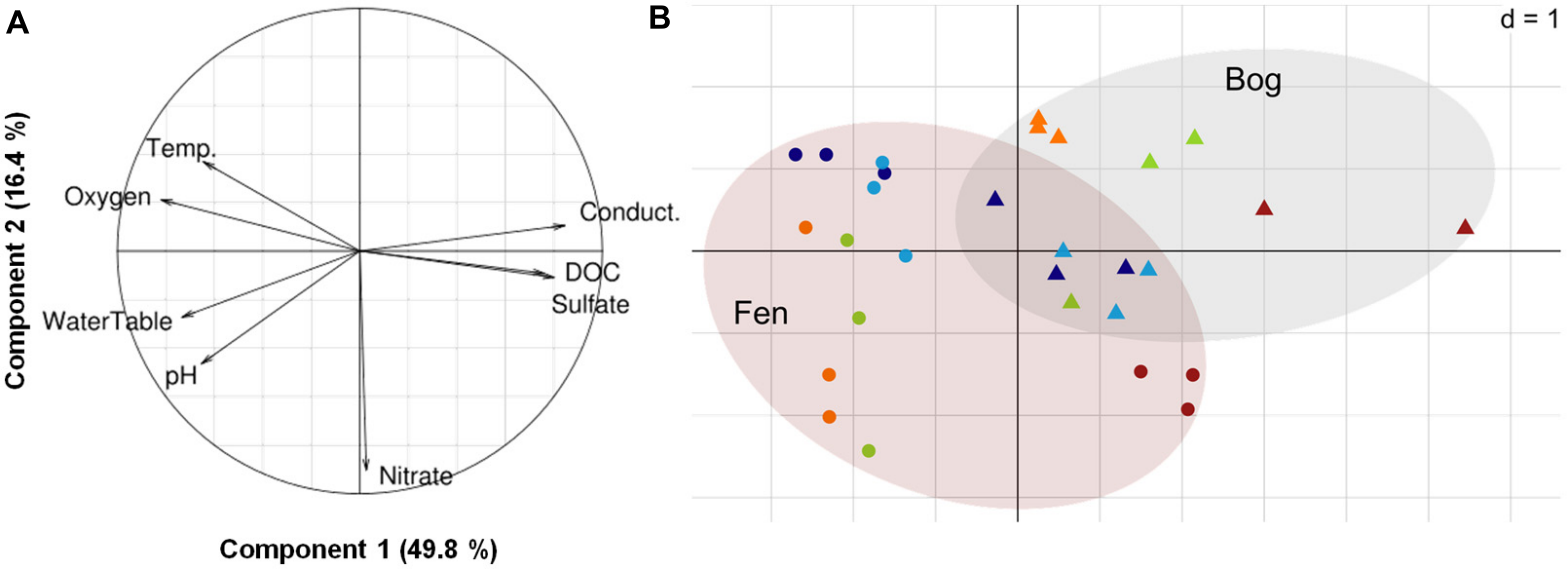
**Principal Component Analysis on the physico-chemical dataset. (A)** Variance explained per component, and correlation circle. **(B)** Projection of the samples. Dot shape represents the habitat (circle: fen, triangle: bog). Dot colors represent the sampling date (dark blue: March 2012, light blue: May 2012, light green: June 2012, orange: July 2012, red: September 2012).

The potential relationships between abiotic and biotic environment and virus communities were analyzed combining fluctuation of physico-chemical variables with viral and prokaryote abundance. The annual mean water-table level was -2.9 ± 4.5 cm in the fen and -18.0 ± 5.0 cm in the bog. Temperature varied more at the upper layer of the bog (annual mean: 11.6 ± 4.4°C) than at the upper layer of the fen (12.4 ± 3.8 °C), but was lower at the lower layer of both stages (fen: 9.3 ± 3.5°C; bog: 9.2 ± 3.5°C) (**Table 1**). We characterized the variation of abiotic physico-chemical parameters with a (PCA) (**Figure 3**). The first component of the PCA accounted for 49.8% of the variance, and was positively correlated with conductivity, sulfate (SO_4_^2-^) and DOC and negatively correlated with water-table level and oxygen (O_2_). The second component accounted for 16.4% of the variance and was mainly correlated with nitrate concentration, which was higher in the fen in June, July, and September. The distribution of data points across the first two components emphasized the differences between fen and bog but also highlighted similar temporal trends within the two development stages, distinguishing the March and May samples from September. The seasonal fluctuations of the water-table were closely linked with variation in water-chemistry, suggesting that water sources and flowpaths affect nutrient concentrations, potentially due to dilution of a limited solute stock (for example, Spearman’s rank correlations; water-table to conductivity: *R* = -0.67, *P* = 6 × 10^-5^, *N* = 29; water-table to sulfate: *R* = -0.66, *P* = 9 × 10^-5^, *N* = 29) (**Table 1**; **Figure 3**). Because the first component represents an integrated variable of spatio-temporal variations of the physico-chemical and hydrological parameters, we used sample coordinates on this axis to model the seasonal abiotic fluctuations for both fen and bog. Log-transformed PA and VPA were both positively, linearly correlated with this abiotic gradient (*N* = 29, PA: *r*^2^= 0.53, *P* < 10^-5^; VPA: *r*^2^= 0.41, *P* < 10^-3^; Supplementary Figure S1), whereas VPR was unrelated to this gradient (*r*^2^= -0.03, *P* = 0.68).

### Viral Community Composition and Diversity

Pyrosequencing of the 12 metaviromes yielded 1 096 889 sequences with an average length of 415 bp (**Table 2**). Analysis revealed that sequences of ribosomal RNA genes accounted for 0.0063% (69 rRNA sequences), which were excluded from subsequent analysis. In addition, the predicted protein coding sequences that matched the functional category databases (KEGG) and the cluster of orthologous genes database (COG) accounted for only 0.38 and 0.45%, respectively. This corresponds to 10-50 times fewer matches than are typically found in conventional short read metagenomes (**Table 2**; Quaiser et al., 2011), indicating a very low level of contamination by genomic DNA from microorganisms (Roux et al., 2013b). This allows the precise characterization of viral diversity and variation in fen and bog through the year.

### Taxonomic Composition

**FIGURE 4:**
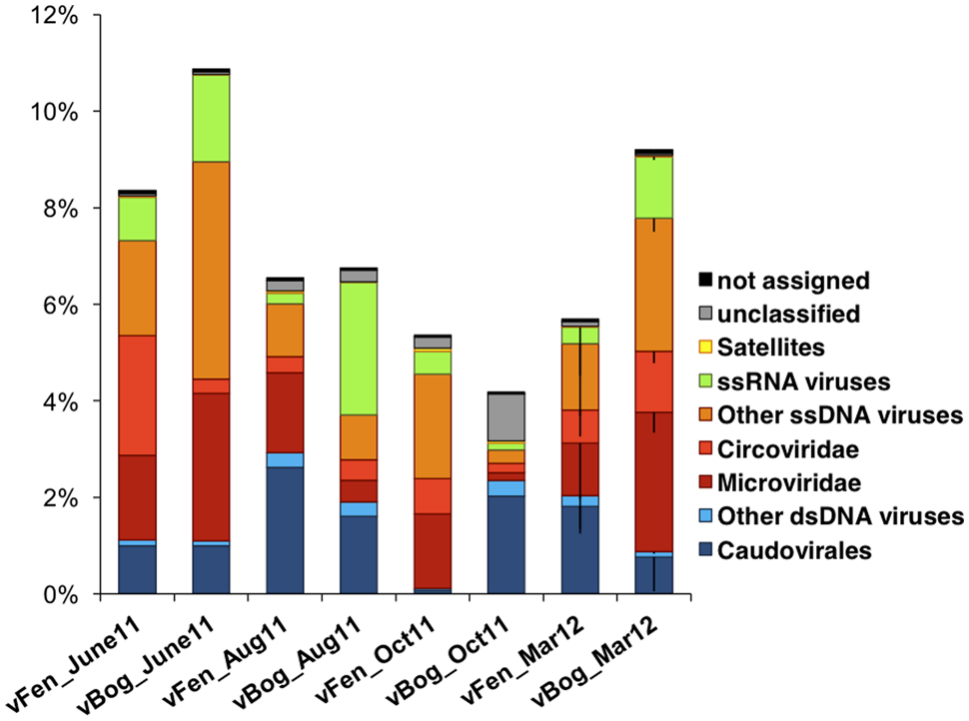
**Relative abundances of the different viral types in the six fen and six bog metaviromes.** Taxonomic affiliations were obtained by tBLASTx against RefSeqVirus (*e*-value 10^-7^). Relative abundances were averaged for March fen and bog metavirome triplicates. Error bars are shown only for negative values and represent standard deviation (*N* = 3).

Sequences were compared against viral genomes from the NCBI RefSeqVirus database. Only a small proportion of sequences, ranging from 4.2% (vBog_Oct11, v = virus/Fen or Bog/sampling date) to 10.9% (vBog_June11) matched the available viral genomes indicating the presence of currently undetected viruses (**Table 2**; **Figure 4**). Matches associated with ssDNA viruses were most common, accounting for a mean of 4.5% of the total number of sequences, with primary assignment to the bacteriophage family *Microviridae* (1.7 ± 1.3%) and to the eukaryal ssDNA family *Circoviridae* (0.9 ± 0.7%). Matches with dsDNA viruses appeared mostly affiliated with the order of *Caudovirales* (1.3 ± 0.8%), which can be hosted by both Bacteria and Archaea. While the protocol was not designed to preserve RNA viruses, we detected sequences matching to ssRNA viruses affiliated with *Tombusviridae* and *Sclerophtora macrospora* virus A representing likely the recently identified so called “chimeric viruses” (Diemer and Stedman, 2012; Roux et al., 2013a). They were present in all samples and accounted for 0.1% (vFen_Aug11) to up to 2.8% (vBog_Aug11) of the total metavirome sequences (**Figure 4**). Nevertheless, the interpretation of these results must be considered with respect to the applied multiple displacement amplification, that was shown to be bias prone towards ssDNA viruses (Kim and Bae, 2011). Due to potential biases no statistical analyses were performed on the proportions of the viral types.

### Genomic Diversity Based Comparisons of the Metaviromes

Due to the lack of virus reference sequences in the databases the majority of sequences (88.3 to 95.4%) remained unaffiliated to known viral taxa. To characterize the remaining, unidentified metaviromes sequences, we analyzed the proportion of similar sequences (four identical kmers of 33 bp) between each pair of metaviromes with Compareads (Maillet et al., 2012). The dendrogram built from the similarity matrix showed two well-separated clusters (Supplementary Figure S2A). One group was composed of metaviromes collected during summer and autumn 2011 (vBog_Aug2011, vFen_Oct11, vBog_Oct2011), while the second group included the communities sampled in winter 2012 (March 2012) and spring 2011 (June 2011) regardless of the peatland development stage.

Sequence comparisons with Compareads provide a global estimate of the proportion of similar sequences without taking into account the internal structure of the sequence sets. Therefore we clustered sequences with a 95% identity threshold to assess the diversity of protein-coding gene sequences and to determine which groups of sequences drive the similarity between metaviromes in the Compareads analysis. Sørensen dissimilarity was calculated for every pair of metaviromes. This distance is based on the distribution (presence-absence) of sequences from the different clusters in the 12 metaviromes. Sørensen dissimilarities between pairwise metaviromes were uncorrelated with the amount of sequences in pairwise comparisons (Spearman’s rank correlation, *R* = 0.08, *P* = 0.495, *N* = 66). The pattern of similarity between the metaviromes supported the first analysis done with Compareads (Supplementary Figure S2B), with nearly identical grouping of summer and autumn communities and winter and spring communities.

**FIGURE 5:**
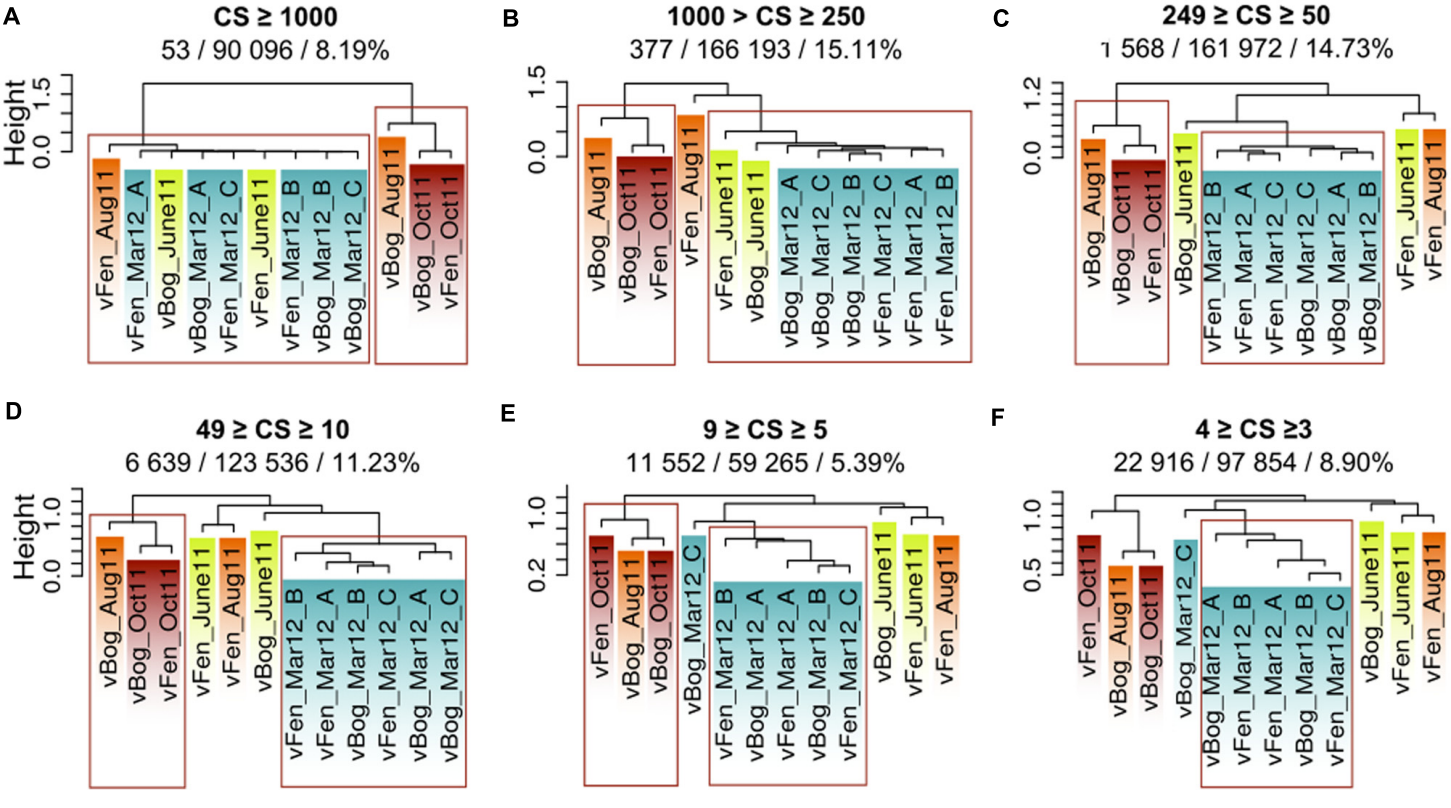
**Metaviromes similarities based on Sørensen Index and on the number of sequences gathered in the clusters. (A-F)** represent different size categories, ranging from the category of clusters that gathered more than 1000 sequences **(A)** to the category of clusters represented by three or four sequences **(F)**. Abbreviations: CS: Cluster Size. Numbers below the class size: No. of clusters represented in the size category / No. of sequences represented in the size category/% of the total metaviromes sequences. Red rectangles represent the most robust clusters (pvclust. au = 0.05).

Unique sequences represented 28% of the total metaviromes and 49% of the sequences belonged to clusters consisting of 10 to more than 1 000 sequences. We split clusters of highly similar sequences into different size categories, depending on the number of sequences they included (**Figure 5**) and computed hierarchical clustering based on Sørensen dissimilarities for each size category. Dendrograms indicated the same contrasted pattern between March viral communities and the summer and autumn group of metaviromes to the exception of vFen_Aug11. This distinction was not significant for clusters smaller than five sequences. This suggests a fundamental change in viral community between the two main groups of metaviromes with low intergroup Sørensen similarity (Supplementary Figure S3). Resemblance between winter and spring metaviromes (Fen and Bog from June 2011 and March 2012) was only significant for clusters larger than 250 sequences. Thus, the resemblance between June and March metaviromes appears to be due to a small number of large clusters.

To analyze the genetic similarities with other metaviromes, we compared the 12 peatland metaviromes with 49 available metaviromes from eight different ecosystem types by hierarchical clustering and tBLASTx (Supplementary Figure S4, see Materials and Methods). Peatland viruses formed a distinct group, clearly separated even from geographically close viral communities originating from freshwater lakes, indicating that these metaviromes represent a unique community characteristic of and structured by its ecosystem (Roux et al., 2012).

### Link Between Viral and Microbial Communities

**FIGURE 6:**
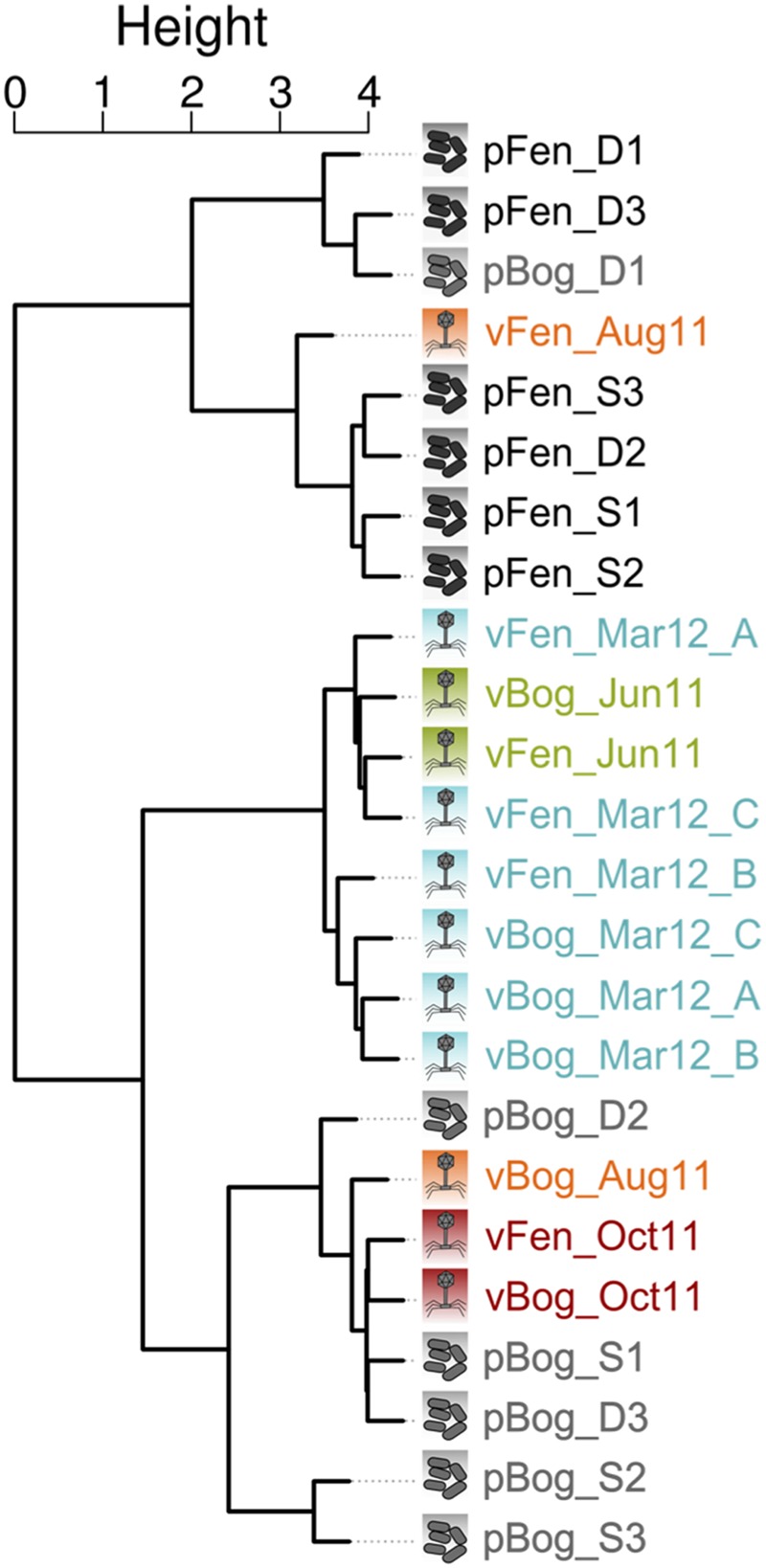
**Hierarchical clustering representing metagenome and metavirome similarity based on sequences shared by at least one metagenome and one metavirome.** Similarities were obtained using Compareads (*k* = 33; *t* = 4). Shared sequences have been clustered using CD-HIT-EST, and a Correspondence Analysis (CA) was performed on the clusters. Hierarchical clustering is based on the two first axes of the CA. Abbreviations: pFen/pBog: metagenome from fen/bog sampled in June 2011; S/D: upper/lower; 1,2,3: metagenome replicates; vFen/vBog: metavirome from fen/bog; (A,B,C): metavirome replicates.

To investigate the interactions between viruses and the microbial communities in *Sphagnum*-dominated peatlands, we sequenced 12 metagenomes from the fen and bog prokaryotic communities (Supplementary Table S2) from the same day and site as the metaviromes vFen_June11 and vBog_June11 (Supplementary Table S1). Metagenome DNA was extracted from the peat matrix allowing finer spatial sampling. In addition, the peat matrix contained the peat pore-water from which the viral particles were sampled. Based on taxonomic affiliations fen and bog prokaryotic communities (hereafter called pFen and pBog) appeared to be predominantly composed of the same main phyla (Supplementary Figure S5). However, regardless of depth, fen and bog appeared to harbor prokaryotic communities with distinct structures as shown by non-metric multidimensional scaling ordination of euclidean distances between metagenomes (Supplementary Figure S6) and with the analysis of similarity (ANOSIM, Euclidean distance, *R* = 0.68, *P* < 0.01). In order to identify viral signatures in these metagenomes, we identified sequences shared by metagenomes and metaviromes using Compareads (four identical kmers of 33 bp). We obtained 18,676 “shared” sequences, of which 30 were from bog metagenomes (pBog), 320 were from fen metagenomes (pFen) (Supplementary Table S2), and 18,326 from the 12 metaviromes (**Table 2**). In most metaviromes, the number of “shared” sequences represented less than 1% of the total sequences but reached up to 12% in vFen_Oct11 (**Table 2**). We clustered sequences with a 95% identity threshold, built a contingency matrix based on the number of sequences from each sample in the different clusters and performed a CA on this contingency matrix. The first two axes of the CA contained 21% of the total information. Hierarchical clustering based on the first two components revealed three groups of samples (**Figure 6**). Two groups included metagenomes sampled in June 2011 from fen and bog, respectively (pFen and pBog_ samples) as well as metaviromes from August and October 2011. The third group contained the metaviromes from June 2011 and March 2012. Thus metaviromes from June 2011 did not cluster with the metagenomes sampled on the same day. Clustering and Sørensen dissimilarity analysis based solely on the subset of metavirome sequences shared with metagenomes revealed the same summer and autumn and winter and spring groupings as obtained for complete metaviromes (Supplementary Figure S7). Among these 18 676 “shared” sequences, a total of 774 sequences (4%) were assigned to references in RefSeqVirus (tBLASTx, *e*-value 10^-7^) indicating that the vast majority (96%) originate from currently unidentified viruses. Most hits were associated with the *Microviridae* subfamily *Gokushovirinae* (ssDNA viruses, 409 hits) and to a lesser extend with the ssDNA viruses *Circoviridae* (173 hits), *Caudovirales* (dsDNA viruses, 134 hits), and *Sclerophtora macrospora* virus A (ssRNA viruses, 19 hits).

## Discussion

### Quality of the Metaviromes

To explore the diversity and potential ecological role of viruses in *Sphagnum*-dominated peatland, we analyzed and compared six fen and six bog metaviromes covering the seasonal periods. The quality of the viromes is essential for comprehensive analysis, since contaminations with microbial genomic DNA would falsify the results. To assure that microbial DNA contamination was satisfactorily low, we applied the pegylation procedure to enrich viral particles (Colombet et al., 2007), DNAse treatment to degrade “free” DNA not protected by capsids, and triplicate whole genome amplification to balance potential amplification bias. The high quality of the virome sequences was shown by the very low abundance of rRNA sequences as well as the low number of matches to functional databases (i.e., KEGG_KO and COG). This is in accordance with the high diversity present in the viral genomic pool and with the high rates of evolutionary changes in viral genomes that are much less conserved than microbial genes (Duffy et al., 2008). Whole genome amplification, as applied here, is known to amplify preferentially circular ssDNA viruses (Kim and Bae, 2011), a bias that cannot be prevented when pooling separate triplicate amplification (Marine et al., 2014). Therefore, the interpretation of the results must be considered with caution. Nevertheless, since all viromes were generated the same way, the inevitably introduced biases should be the same for all allowing reliable comparative analysis.

It has been hypothesized that viruses infecting eukaryotes might be more important in terrestrial ecosystems and wetlands, where protozoan and fungal biomass is higher, while bacteriophages dominate viral consortia in marine and freshwater ecosystems (Farnell-Jackson and Ward, 2003; Jackson and Jackson, 2008; Kimura et al., 2008). Concerning the peatland metaviromes, among the sequences that matched viral genome databases, similar proportions of sequences were assigned to eukaryotic viruses, such as *Circoviridae* and *Sclerophtora macrospora* virus A-like viruses, and to prokaryotic viruses, such as *Caudovirales* or *Microviridae*. However, due to the vast majority of sequences being unassigned and to the high variability of viral genes, it remains impossible to determine whether viral communities in peatlands are dominated by prokaryote or eukaryote infecting viruses.

### Successional Patterns of Viral and Microbial Communities

Ecological integration of the viral compartment into ecosystem functioning is mainly obtained through approaches combining virome sequences and viral abundance analysis (Wommack et al., 2015). In order to characterize viral ecology of *Sphagnum-*dominated peatlands, we monitored seasonal abundance and diversity of viruses and prokaryotes at two different depths of fen and bog and attempted to identify whether these were correlated to abiotic factors. Fens and bogs are development stages of peatlands that differ fundamentally in vegetation (*Sphagnum* and vascular plants) and associated physico-chemistry (Rydin and Jeglum, 2006). Thus, as already observed for microbial diversity (Opelt et al., 2007; Bragina et al., 2012), we hypothesized that peatland development stage and associated *Sphagnum* habitat would be the major driver in the distribution of virus and prokaryote abundance and diversity. Our results confirm that the physico-chemical conditions and the structure of the prokaryotic communities differ between the 2 dynamic stages, but, surprisingly, we did not observe any significant difference in VPA, PA, and viral diversity between fen and bog. The viral communities showed no systematic spatial trend and high variability even within replicates.

While we did not detect any significant spatial differences, we observed a significant seasonal fluctuation of virus diversity and abundance. For both fen and bog, VPA and PA (log-transformed data) were strongly correlated with the seasonal fluctuations of water-table, DOC, conductivity and sulfate: i.e., VPA and PA were higher when water-table was low and DOC and sulfate were high (Supplementary Figure S1). DOC has been recognized as a key factor in the C-balance of *Sphagnum-*dominated peatlands (Billett et al., 2004) and its patterns are driven by both biological activity (microbial production and consumption, plant exudation) and abiotic variables such temperature, water-table level or acidity (Clark et al., 2009) with seasonal fluctuations as a consequence (Moore, 1987). While the pH is recognized as an integrated physico-chemical variable, we did not register strong influence on PA and VPA. The increased pH observed during June and July in the fen is potentially due to photosynthetic activity with strong assimilation of dissolved inorganic carbon by microalgae, which significantly develop in submerged *Sphagnum*-fen at the beginning of summer (Gilbert et al., 1998). As viral activity is dependent on bacterial production (Middelboe, 2000), the correlations between DOC and VPA and PA suggest a net production of DOC with increasing microbial activity including viral lysis. DOC concentrations also depend on temperature and water flows, which play a key role in the production and redistribution of carbon in the peat (Waddington and Roulet, 1997; Clark et al., 2009), the two factors interacting during drawdown and flooding periods. However, we did not evidence a clear combination of DOC and temperature. The fluctuations of the water-table depend largely on hydrologic inputs that are rainfall in the bog and mainly runoff and groundwater inflow in the fen, which occur at the yearly scale (seasons) and at a short-time scale (episodic events). Sulfate also interacts with DOC concentrations, especially during drought periods when significant production of sulfate may increase peat acidification and ionic strength (Clark et al., 2009). In our study, sulfate concentrations were significantly higher during water-table drawdowns and were positively linked to DOC. In addition, we could not show whether the production of sulfate was linked to nitrate through microbial sulfur oxidation as already demonstrated in peatlands (Burgin and Hamilton, 2008). Nitrate seems to play a key role only during summer in the fen (see component 2 of the ACP), a dynamic stage in which nitrogen mineralization significantly occurs at this season (Francez and Loiseau, 1999). Our results suggest that viruses and prokaryotes are more abundant at the lower surface layer regardless of peatland development stage. This is in accordance with a previous study concerning prokaryote abundance (Dedysh et al., 2006) and potentially due to more buffered temperature and water-table fluctuations at the lower layer providing more stable conditions. Altogether, these results indicate that seasonal changes in temperature and precipitations (allogenic variables) influence PA and VPA *via* water-table fluctuations and consequently nutrient concentrations with larger effects at the upper than at the lower layer.

Comparisons of metaviromes with metagenomes showed that temporal variations are more influential than differences in peatland habitat in structuring viral communities. There was a particularly substantial shift in sequence composition from spring to autumn with distinct patterns of composition and abundance, suggesting the existence of ecological succession of viral communities at the seasonal scale. This pattern appears to be consistent inter-annually, with metaviromes sampled about a year apart (June 2011 and March 2012) clustering together. These findings suggest, for the first time, that a cyclic succession in peatlands affects free-occurring viruses at the community level. Recent studies on marine ecosystems also described seasonal fluctuations of viral communities at the ocean surface (Chow and Fuhrman, 2012; Pagarete et al., 2013). In these studies, which focused on the diversity of a viral gene marker, seasonality was mainly characterized by fluctuations of dominant viral types while in our study, seasonality was associated with a general change in the composition of viral communities.

### Viruses and Carbon Cycling in Sphagnum-Dominated Peatlands

Viruses are believed to be key components of the carbon cycle in many ecosystems, both altering carbon fluxes and contributing to C-redistribution through bacterial lysis (Fuhrman, 1999; Middelboe and Lyck, 2002; Ankrah et al., 2014). Despite recent analyses of peatland microbial food-webs (Lamentowicz et al., 2013), the significance of viruses in the functioning of *Sphagnum*-dominated peatlands remains unknown.

In viral ecology, VPR is generally considered as an indicator of the bacterial hosts metabolic state (Williamson et al., 2005; Kimura et al., 2008) because viral burst size, and thus viral abundance is positively correlated with microbial growth rate (Middelboe, 2000). In the studied peatland, the VPR was low and did not differ between fen and bog, despite the differences between dead organic matter produced in the two dynamic stages, in relation to the dominant *Sphagnum* species (Francez, 1995; Thormann et al., 2003). The low VPR compared with other ecosystems is likely due to lower metabolic activity of microorganisms, that is in accordance with the functioning of *Sphagnum-*dominated peatlands where decomposition is slowed down due to constraining conditions (Rydin and Jeglum, 2006; Artz, 2009) and the presence of a significant proportion of dormant cells in the community (Dedysh et al., 2006; Pankratov et al., 2011).

We detected a VPR peak in June 2011, just before a broad modification of viral community composition in fen and bog. Viruses interact with their hosts through at least two main strategies: the lytic and the lysogenic life cycles, the latter is believed to be favored when microbial activity is low (Danovaro et al., 2002; Payet and Suttle, 2013; Sime-Ngando, 2014). The change in the viral community composition in summer could result from a seasonal shift in the active part of the microbial community and related C-cycling processes *via* decomposition that show seasonal patterns (Basiliko et al., 2005; Sun et al., 2012). This illustrates a transition from lysogenic to lytic strategies of the viruses infecting the newly active prokaryotes. This hypothesis is supported by the low PA associated with the VPR peak in June 2011, which could result from virus-mediated bacterial lysis, and by the similarities between spring metagenomes (June) and summer and autumn viromes, suggesting the presence of prophages in the microbial genomes in June, that were later released and detected in the metaviromes in August and October.

## Conclusion

We applied an integrated approach linking virome sequence analysis, viral particle and prokaryote abundance, physico-chemical parameters and metagenome-virome comparison to get insights into the ecological functioning of the viral community in peatlands. We found that viral community abundance and diversity in *Sphagnum-*dominated peatlands express an ecological succession, and that viruses, as well as their hosts, are strongly influenced by the temporal fluctuations at the peatland surface. The observed low VPR, compared with other ecosystems, is in accordance with slowed down decomposition processes in *Sphagnum-*dominated peatlands. The observed shift in viral diversity suggests a seasonal change of the microbial community and the associated switch of viral life-cycle strategies during summer and autumn highlighting the importance of virus-host interactions as they control the dynamics of microbial communities. These patterns may be related to changes in C-cycling processes but further studies are needed to strictly link microbial and viral diversity with C-transformations in the peat. These should focus on the seasonality of viruses that infect different kind of hosts (prokaryotes/eukaryotes) in order to identify the main factors driving this succession and changes in functioning processes, as already suggested for seasonal fluctuations of plankton (Sommer et al., 2012).

To assess the ecological relevance of the diversity of viral communities, analysis of the spatio-temporal dynamics of ecosystem-specific metaviromes as applied here, rather than cross-biomes comparisons, represent a powerful approach to overcome the lack of viral genomes in the databases, and to take advantage of the whole diversity carried in the sequenced viral communities.

## Conflict of Interest Statement

The authors declare that the research was conducted in the absence of any commercial or financial relationships that could be construed as a potential conflict of interest.
