# Supplementary material for: Dynamics of Viral Abundance and Diversity in a Sphagnum-Dominated Peatland: Temporal Fluctuations Prevail Over Habitat
Source: Front Microbiol. 2016 Jan 6;6:1494. doi: 10.3389/fmicb.2015.01494 (PMC4701944; doi:10.3389/fmicb.2015.01494)
Supplement: Supplementary file 1 [file Supplementary_Tables.PDF]

Supplementary Tables

Dynamics of viral abundance and diversity in a *Sphagnum*-dominated peatland: temporal fluctuations prevail over habitat

Flore Ballaud[1], Alexis Dufresne[1], André-Jean Francez[1], Jonathan Colombet[2], Télesphore Sime-Ngando[2] and Achim Quaiser[1]

[1]Université de Rennes 1, UMR CNRS 6553 ECOBIO, Campus de Beaulieu, 35042 Rennes, France

[2] Université Clermont Auvergne, Université Blaise Pascal, BP 10448, F-63000 Clermont-Ferrand, France. CNRS, UMR 6023, Laboratoire Microorganismes: Génome et Environnement (LMGE), 24 avenue des Landais, F-63171 Aubière, France

Correspondence: Achim Quaiser, ECOBIO, Université de Rennes 1, Campus de Beaulieu, 263 avenue du Général Leclerc, 35042 cedex Rennes, France. Building 14A, office 222. Tel.: +33 2 23 23 53 51 37. E-mail: Achim.Quaiser@univ-rennes1.fr

Table S1. Detailed information about peat samples.

		Abundance (VPA, PA)	Physico-chemistry	Metaviromes	Metagenomes
**Year**	**Month**	No. of samples (fen/bog; upper/lower)	No. of samples (fen/bog)		
2010	May	4			
2011	June	4		vFen_June11, vBog_June11	pFen_S (1, 2, 3), pFen_D (1, 2, 3), pBog_S (1, 2, 3), pBog_D (1, 2, 3)
	Aug_A	4		vFen_Aug11, vBog_Aug11	
	Aug_B	4			
	October	4		vFen_Oct11, vBog_Oct11	
2012	March		2	vFen_Mar12 (A, B, C) vBog_Mar12 (A, B, C)	
	May		2		
	June	4	2		
	July	4	2		
	August		2		
	September	4	2		
	November		2		
	Total samples	32	14	8	4
	Triplicates	96	42	12 (2 samples in triplicate)	12

Upper: 5 to 10cm below the active *Sphagnum* layer; Lower: 10 to 15cm below the active *Sphagnum* layer.

Table S2. Main characteristics of the 6 fen and 6 bog metagenomes.

	pFen_S1	pFen_S2	pFen_S3	pFen_D1	pFen_D2	pFen_D3	pBog_S1	pBog_S2	pBog_S3	pBog_D1	pBog_D2	pBog_D3
**Depth***		**Upper**			**Lower**			**Upper**			**Lower**	
**No. of sequences**	93 863	204 336	9 963	176 881	11 987	333 485	2 091	19 844	25 841	9 543	3 851	1 542
**Sequence average size (bp)**	454	455	452	455	452	455	452	452	455	456	454	450
**Total nucleotides (Mbp)**	42.67	93.04	0.45	80.64	5.43	152.05	0.95	8.99	11.77	4.36	1.75	0.7
**% matches in database**	54.00%	52.00%	52.00%	52.00%	53.00%	53.00%	60.35%	57.14%	56.13%	53.86%	54.09%	57.20%
****Matches to prokaryotes**	98.49%	99.39%	96.83%	99.01%	94.14%	99.42%	92.27%	94.19%	96.76%	93.53%	96.71%	95.81%
****Matches to viruses**	0.02%	0.01%	0.02%	0.01%	0.01%	0.01%	0.05%	0.01%	0.00%	0.00%	0.00%	0.00%
****Matches to eukaryotes**	1.37%	0.52%	2.99%	0.87%	5.76%	0.46%	7.56%	5.73%	3.19%	6.40%	3.29%	4.19%
**No. of shared virome sequences*****	67	73	17	78	7	78	1	14	10	3	1	1
**% of shared virome sequences**	0.07%	0.04%	0.17%	0.04%	0.06%	0.02%	0.05%	0.07%	0.04%	0.03%	0.03%	0.06%

*Upper: 5 to 10 cm below the *Sphagnum* capitula layer; Lower: 10 to 15 cm below the *Sphagnum* capitula layer;
**Relative abundances are given as a proportion of the sequences that matched against NCBI “nr” protein database;
***Shared: sequences that are common between at least a metagenome and a metavirome (Compareads1.2.2, t=4, k=33).
