# Supplementary material for: Dynamics of Viral Abundance and Diversity in a Sphagnum-Dominated Peatland: Temporal Fluctuations Prevail Over Habitat
Source: Front Microbiol. 2016 Jan 6;6:1494. doi: 10.3389/fmicb.2015.01494 (PMC4701944; doi:10.3389/fmicb.2015.01494)
Supplement: Supplementary file 2 [file Supplementary_Figures.PDF]

Supplementary Figures

Dynamics of viral abundance and diversity in a *Sphagnum*-dominated peatland: temporal fluctuations prevail over habitat

Flore Ballaud[1], Alexis Dufresne[1], André-Jean Francez[1], Jonathan Colombet[2], Télesphore Sime-Ngando[2] and Achim Quaiser[1]

[1]Université de Rennes 1, UMR CNRS 6553 ECOBIO, Campus de Beaulieu, 35042 Rennes, France

[2] Université Clermont Auvergne, Université Blaise Pascal, BP 10448, F-63000 Clermont-Ferrand, France. CNRS, UMR 6023, Laboratoire Microorganismes: Génome et Environnement (LMGE), 24 avenue des Landais, F-63171 Aubière, France

Correspondence: Achim Quaiser, ECOBIO, Université de Rennes 1, Campus de Beaulieu, 263 avenue du Général Leclerc, 35042 cedex Rennes, France. Building 14A, office 222. Tel.: +33 2 23 23 53 51 37. E-mail: Achim.Quaiser@univ-rennes1.fr

Figure S1

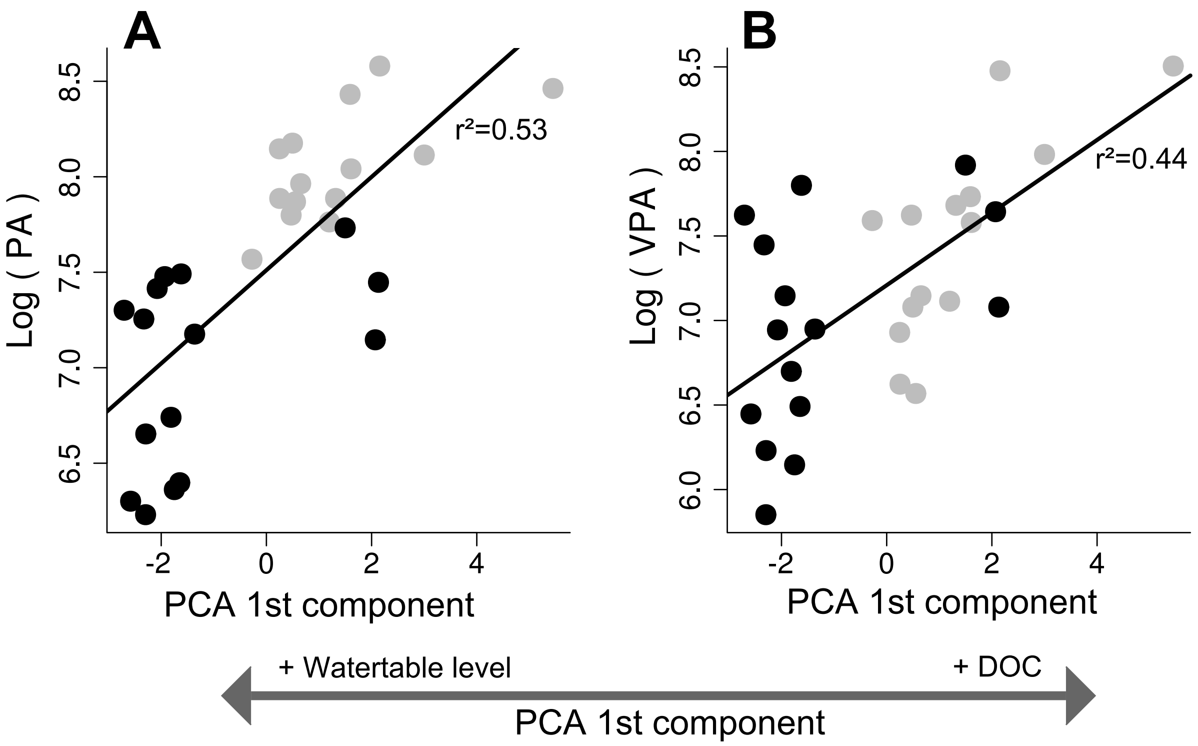

Figure S1. Linear regressions between log-transformed PA (A), VPA (B) and the abiotic gradient. The abiotic gradient corresponds to the data points' position on the 1st component of the PCA. Black dots: fen samples. Grey dots: bog samples.

Figure S2

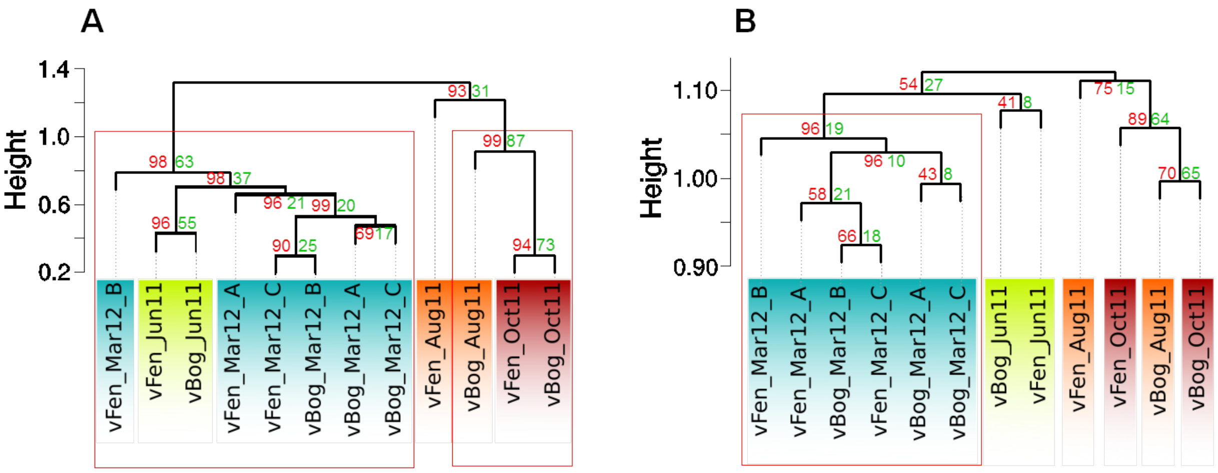

Figure S2. Metavirome similarity based on Compareads analysis **(A)** and Sørensen index dissimilarity **(B)**. Compareads analysis is based on the amount of identical sequences between pairwise samples (k=33, t=4). Sørensen index is based on the presence/absence of sequences from each metaviromes in the different clusters. Red rectangles represent the most robust groups obtained following hierarchical clustering (pvclust, au=0.05).

Figure S3

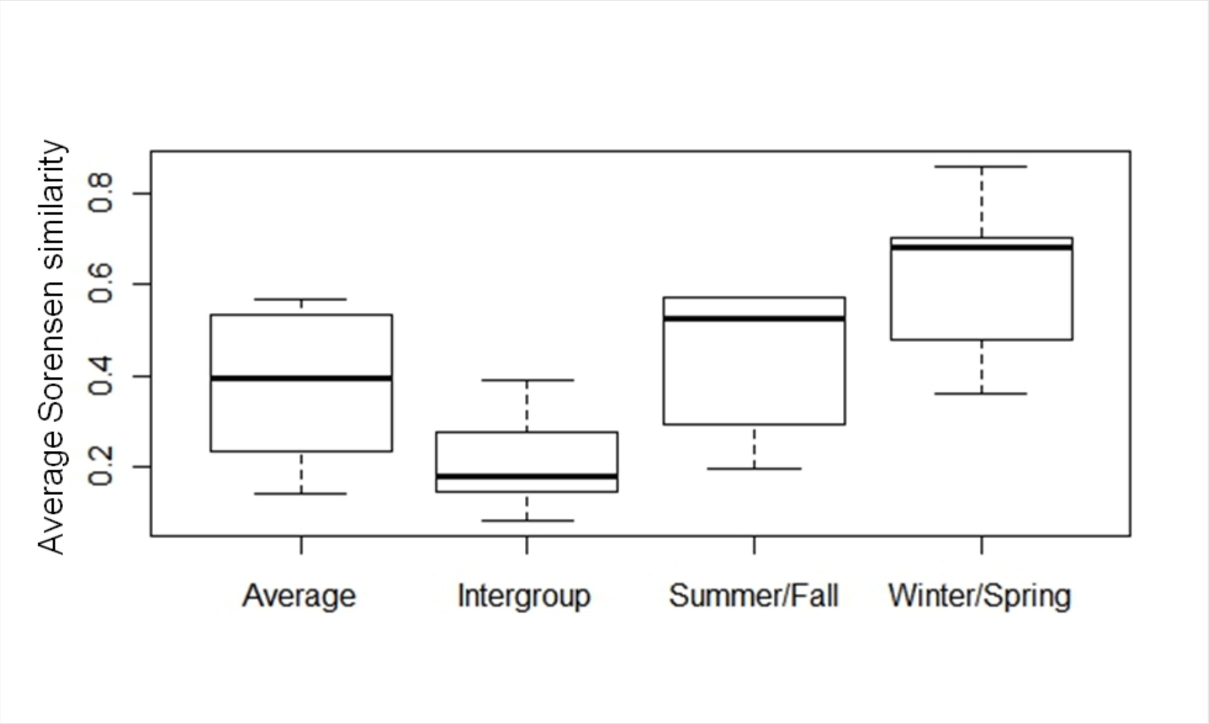

Figure S3. Average Sørensen similarity for the different summer/autumn and winter/spring groups of metaviromes. The groups were determined using the most robust clusters from Figure 5. For each size category, except the last one (4 ≤ CS ≤ 3, only one robust group), average Sørensen similarity (1- Sørensen dissimilarity) was calculated among the metaviromes from each group, and between metaviromes that originated from different groups (Intergroup). Values for each group from each size category were averaged corresponding to the average similarity between all the metaviromes, independently of their assignation to a group.

Figure S4

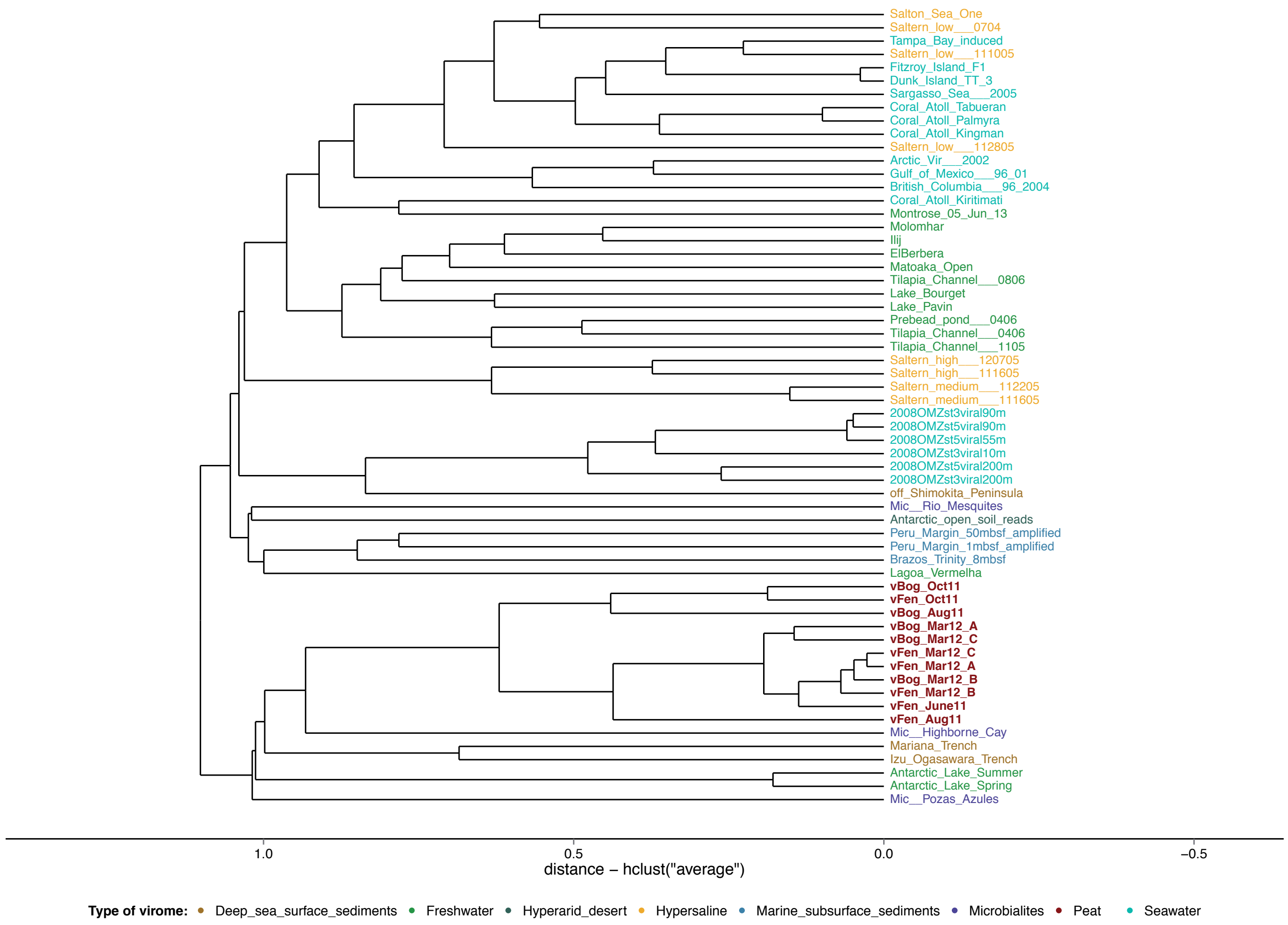

Figure S4. Hierarchical clustering of the peatland metaviromes and metaviromes available for different types of ecosystems (Metavir blast-based comparison). Peatland metaviromes (dark red) form one group. Lake Bourget and lake Pavin are two freshwater lakes (green) geographically close to the peatland. The viral communities from these two lakes strongly differ from the peatland communities. The difference between the communities from the two lakes is equivalent to the difference between the peatland summer/autumn and winter/spring communities.

Figure S5

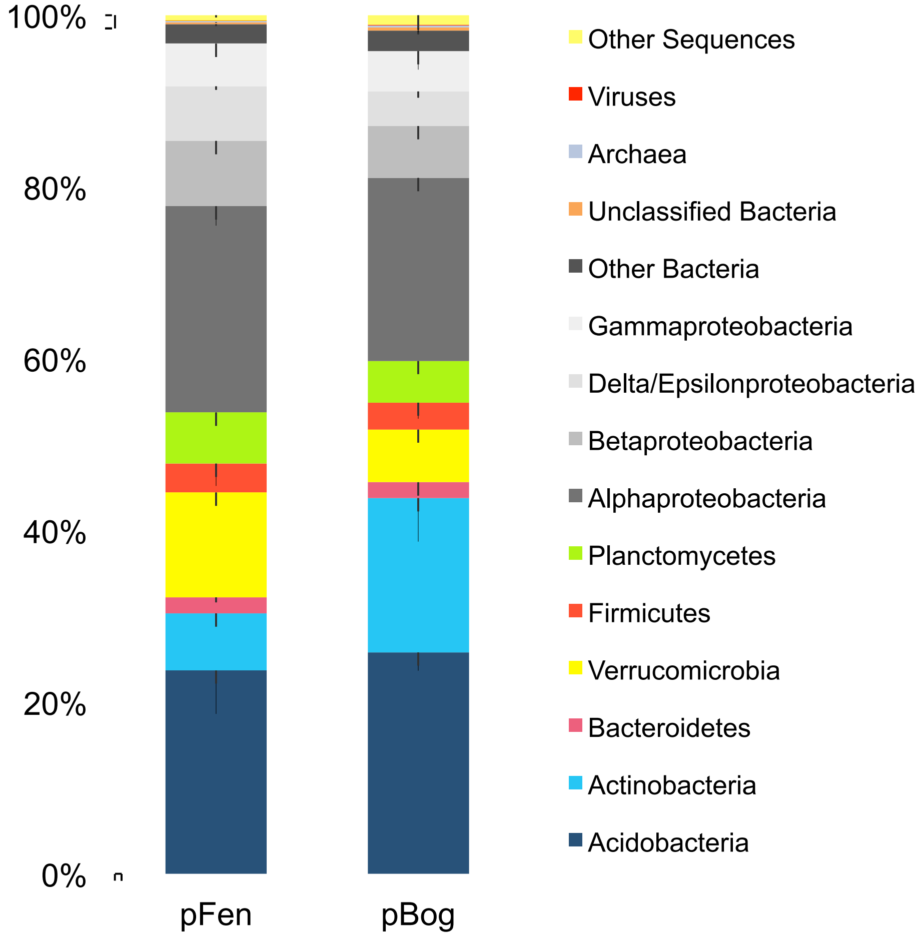

Figure S5. Relative abundance of the prokaryotic phyla in fen and bog microbial metagenomes based on tBLASTx assignations against NCBI non-redundant protein database (relative proportions of assigned sequences). Number of sequences fen: 830515; number of sequences bog: 62712. Error bars represent the negative value of the standard deviation (N=6).

Figure S6

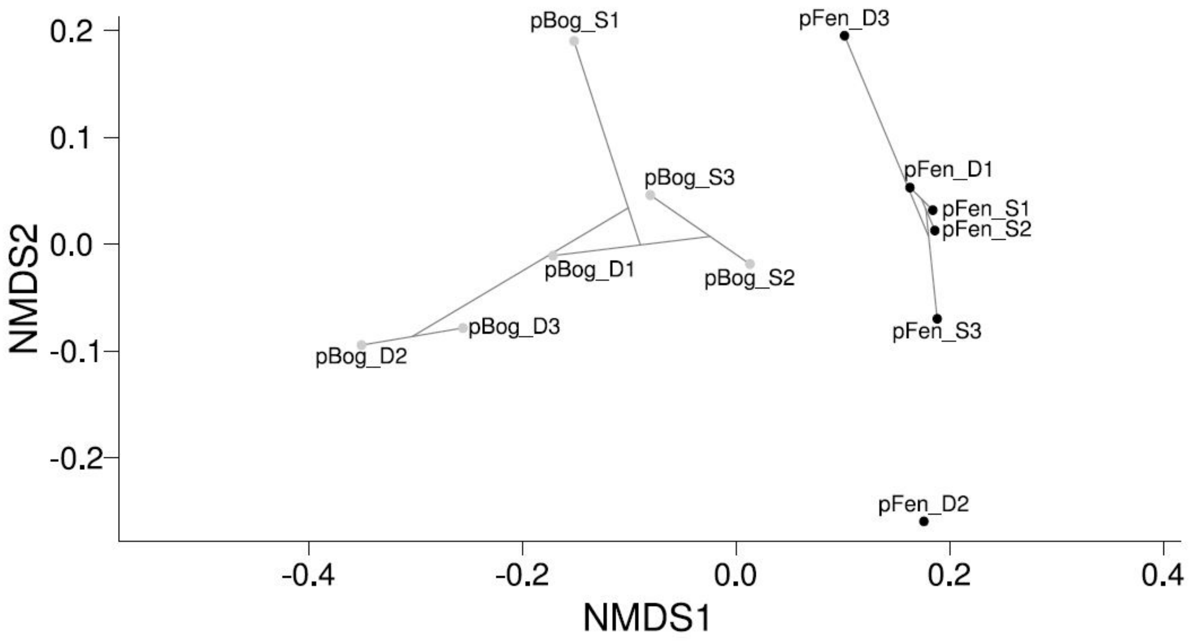

Figure S6. NMDS representing fen and bog microbial communities depending on the euclidean distance between each sample at the phyla level. Stress= 0.03.

Figure S7

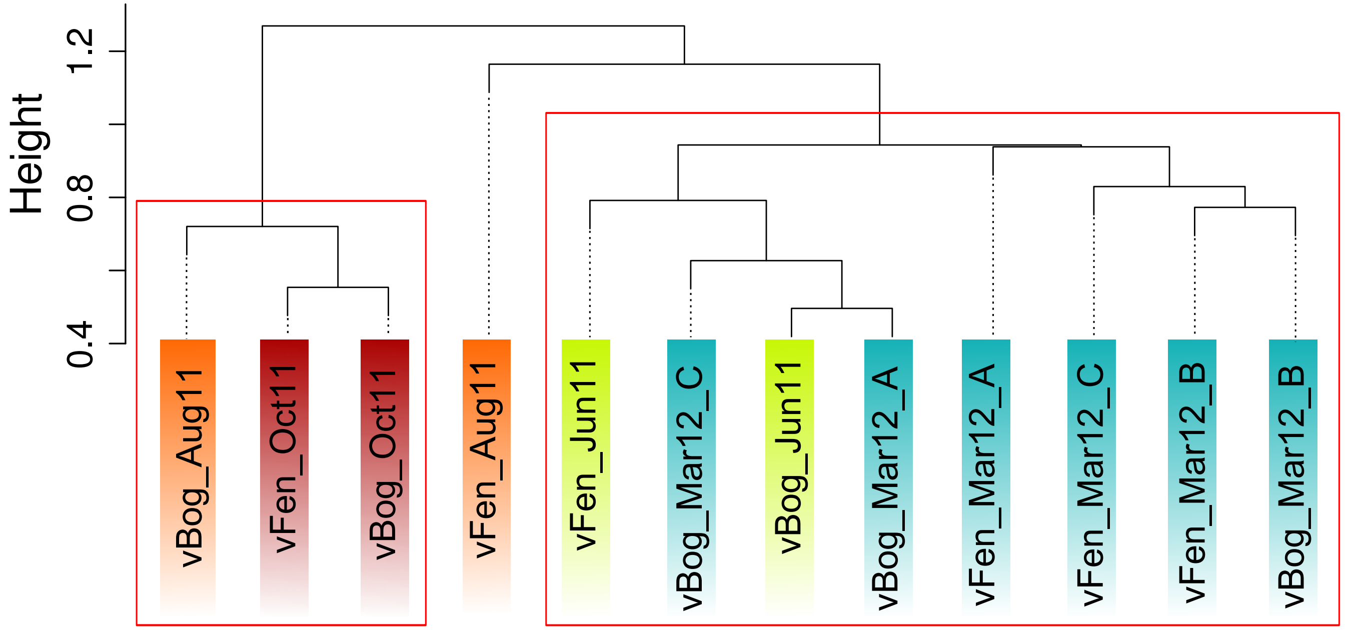

Figure S7. Hierarchical clustering of the metaviromes sequences shared with the metagenomes based on Sørensen dissimilarity (18 326 sequences retrieved with Compareads1.2.2, k=33. t=4). Sequences have been clustered using CD-HIT-EST and Sørensen dissimilarity index was calculated between pairwise metaviromes. Red rectangles represent the most robust clusters (pvclust, au=0.05).
